# LncTarD: a manually-curated database of experimentally-supported functional lncRNA–target regulations in human diseases

**DOI:** 10.1093/nar/gkz985

**Published:** 2019-11-12

**Authors:** Hongying Zhao, Jian Shi, Yunpeng Zhang, Aimin Xie, Lei Yu, Caiyu Zhang, Junjie Lei, Haotian Xu, Zhijun Leng, Tengyue Li, Waidong Huang, Shihua Lin, Li Wang, Yun Xiao, Xia Li

**Affiliations:** 1 College of Bioinformatics Science and Technology, Harbin Medical University, Harbin 150081, China; 2 College of Bioinformatics, Hainan Medical University, Haikou 570100, China

## Abstract

Long non-coding RNAs (lncRNAs) are associated with human diseases. Although lncRNA–disease associations have received significant attention, no online repository is available to collect lncRNA-mediated regulatory mechanisms, key downstream targets, and important biological functions driven by disease-related lncRNAs in human diseases. We thus developed LncTarD (http://biocc.hrbmu.edu.cn/LncTarD/ or http://bio-bigdata.hrbmu.edu.cn/LncTarD), a manually-curated database that provides a comprehensive resource of key lncRNA–target regulations, lncRNA-influenced functions, and lncRNA-mediated regulatory mechanisms in human diseases. LncTarD offers (i) 2822 key lncRNA–target regulations involving 475 lncRNAs and 1039 targets associated with 177 human diseases; (ii) 1613 experimentally-supported functional regulations and 1209 expression associations in human diseases; (iii) important biological functions driven by disease-related lncRNAs in human diseases; (iv) lncRNA–target regulations responsible for drug resistance or sensitivity in human diseases and (v) lncRNA microarray, lncRNA sequence data and transcriptome data of an 11 373 pan-cancer patient cohort from TCGA to help characterize the functional dynamics of these lncRNA–target regulations. LncTarD also provides a user-friendly interface to conveniently browse, search, and download data. LncTarD will be a useful resource platform for the further understanding of functions and molecular mechanisms of lncRNA deregulation in human disease, which will help to identify novel and sensitive biomarkers and therapeutic targets.

## INTRODUCTION

Studies have shown that >98% of the human genome does not encode protein sequences. In particular, long non-coding RNAs (lncRNAs) are a class of important non-coding RNAs with lengths >200 nt (1). With the rapid development of both experimental technology and computational methods, the number of lncRNAs has increased drastically over the past few years. Recent advances in lncRNA-related research concerning their sequence, expression, and function have led to the creation of several publicly available lncRNA resources. For example, LNCipedia 5 (lncRNA sequence and annotation) (2), LNCediting (functional prediction of RNA editing in lncRNAs) (3), lncRNASNP2 (single nucleotide polymorphisms in lncRNAs) (4), lncRNome (lncRNA annotation) (5), lncRNAtor (lncRNA sequence, annotation, gene expression, protein binding and conservation) (6) and lncRNAdb v2.0 (lncRNA sequence, expression data and the literature) (7) all focus on the basic information and functional characteristics of lncRNAs. LncRNA2Target v2.0 (predicted lncRNA–target relationships) (8), ChIPBase v2.0 (predicted TF-ncRNA regulations) (9), LncReg (experimentally validated lncRNA-associated regulatory entries) (10), and DIANA-LncBase v2 (experimentally verified and predicted miRNA-lncRNA pairs) (11) focus on lncRNA-associated regulatory entries without disease information. More importantly, the involvement of lncRNAs in numerous human diseases, especially in cancers, has received a great deal of attention. Recently, experimentally-supported or predicted lncRNA–disease associations have been stored in publicly available databases, such as LncRNADisease 2.0 (experimentally-supported and predicted lncRNA–disease associations) (12) and Lnc2Cancer v2.0 (experimentally supported lncRNA-cancer associations) (13). However, important information concerning key downstream targets, biological functions driven by disease-related lncRNAs, as well as lncRNA-dependent mechanisms of gene expression deregulation in human diseases still remain hidden in a large amount of dispersed literature, and have yet to be extensively and systematically collected.

Indeed, anomalies in lncRNA-dependent gene-regulatory mechanisms contribute to the occurrence and progression of human diseases. For instance, the lncRNA HOTAIR could be involved in the downregulation of HOX loci through H3K27 trimethylation, thereby regulating breast invasion and metastasis by binding to polycomb repressive complex 2 (PRC2) (14). In addition to the epigenetic regulation of genes, several lncRNAs could act as ceRNAs by sponging miRNAs to reduce their inhibitory effect on their target protein-coding mRNAs, thus participating in post-transcriptional processing. For example, SNHG16 promotes epithelial-to-mesenchymal transition (EMT) in esophageal squamous cell carcinoma (ESCC) by competing with miR-140-5p to positively regulate its key target ZEB1 (15,16). Understanding the precise molecular mechanisms by which lncRNA regulates downstream key mediators to affect important biological functions is necessary to help explain the roles of lncRNAs in the pathogenesis of human diseases and will be a critical first step in exploring these potential new avenues in cancer therapy. However, such important information concerning how these disease-related lncRNAs contribute to pathogenesis, which targets are the downstream key mediators, and which functions are affected has been largely ignored.

To fill this gap, we therefore developed LncTarD, a novel database that collects and integrates disease-associated lncRNA–target regulations into a comprehensive resource for the first time (Supplementary Table S1). The current version of LncTarD includes 2822 disease-associated lncRNA–target regulations, including 1613 experimentally-supported functional regulations and 1209 expression associations, involving 475 lncRNAs and 1039 targets contributing to 140 biological functions in 177 human diseases. All disease-associated lncRNA–target regulations in LncTarD are experimentally supported and manually curated from the published literature. Additional important information, including lncRNA-mediated regulatory mechanisms, important biological functions, and drug response-related lncRNA–target regulations, are also provided. A large amount of TCGA pan-cancer transcriptome data was further integrated into LncTarD to help characterize the functional dynamics of key lncRNA–target regulations. With the rapidly increasing interest in lncRNA regulations, we hope that LncTarD can serve as a timely and valuable resource to facilitate the understanding of the functions and regulatory mechanisms of lncRNA deregulation in the pathogenesis of human disease.

## DATA COLLECTION, PROCESSING AND IMPLEMENTATION

**Figure 1.**
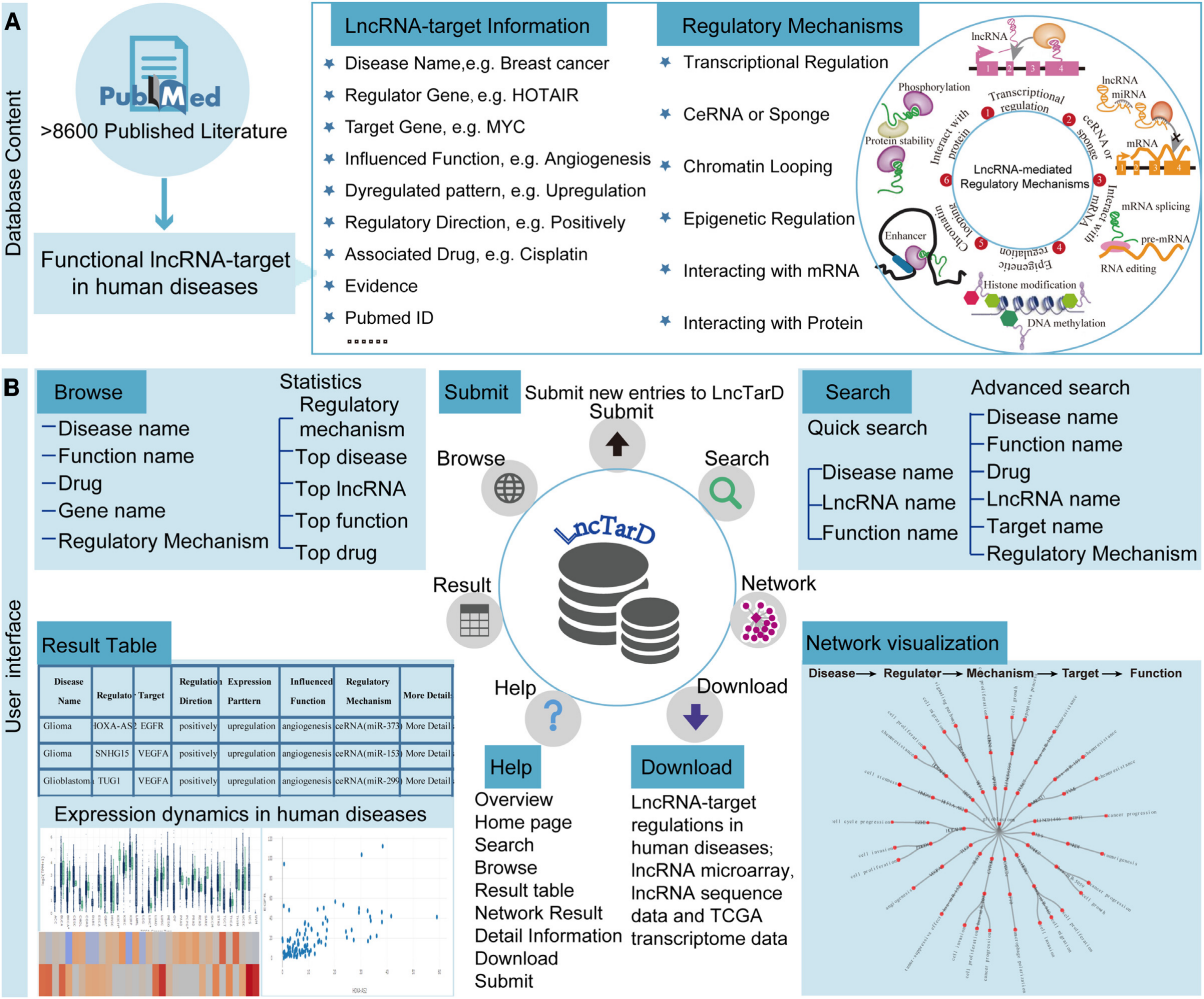
Overview of disease-associated lncRNA–target collection, annotation and LncTarD database features. (**A**) Manually-curated functional lncRNA–target regulations in human diseases in LncTarD; (**B**) browse, search, result, and network modules for disease-associated lncRNA–target regulations.

First, we searched the PubMed literature database using the master keywords ‘long noncoding RNA,’ ‘lncRNA’ and ‘lincRNA,’ in combination with ‘cancer,’ ‘tumor,’ ‘disease,’ ‘carcinoma,’ ‘dysfunction,’ ‘proliferation,’ ‘invasiveness,’ ‘invasion,’ ‘migration,’ ‘patient,’ ‘patients,’ ‘abnormalities,’ ‘syndrome,’ ‘disorder’ and ‘human.’ PubMed articles were identified by searching these keywords in full text on PubMed. We then manually screened the abstracts in order to extract the entries that described associations between lncRNA–target regulations and human diseases from 8866 PubMed publications (17). Next, the full text of these articles was double-reviewed to extract the detailed lncRNA-mediated regulatory mechanisms in human diseases. Only the articles meeting all of the following criteria were retained: (i) lncRNAs associated with human diseases; (ii) identification of key target genes of the lncRNA and (iii) mention of important biological functions affected by lncRNA–target regulations. Through strict screening, 1862 articles comprised relevant information that was incorporated into the database. Second, we retrieved the lncRNA, target and disease names, direction of regulation (positively-F/E or negatively-F/E), experimental method for each interaction, expression pattern of lncRNA (upregulated or downregulated), experimental method and lncRNA microarray, lncRNA sequence data for lncRNA dysregulation, lncRNA coordinates, positively- or negatively-influenced biological functions emphasized by the original reference articles, lncRNA-mediated regulatory mechanisms (such as ceRNA or sponge, and epigenetic regulation), and a brief description of functional lncRNA–target regulation in human diseases from the original studies and reference information. Notably, we extracted and recorded the cancer subtype information from the PubMed literature. We used negatively-F (or positively-F) and negatively-E (or positively-E) to represent negatively (or positively) affecting function and expression, respectively. For example, lncRNA:miRNA sponge interactions are annotated as ‘negatively-F,’ which potentially means that miRNA function (not expression) is negatively affected. In other regulations, ‘negatively-E’ would mean the reduction of target expression. Only high-quality functional regulations with strong experimental evidence such as RNA pull-down assay, RNAi, qRT-PCR, and luciferase reporter assay were selected. Our database also offers the association between the expression levels of disease-associated lncRNA and associated target genes. Additionally, associations between cancer hallmarks and lncRNA–target regulations were obtained by semantic similarity using GOSemSim package. A lncRNA–target was considered to be related to a cancer hallmark if the semantic similarity score was >0.8. Third, all selected functional lncRNA–target regulations in human diseases were rechecked for the lncRNA as well as target and disease names, with some names being replaced with official or recommended names. If the lncRNAs were not identical to the Ensembl entries, we used the lncRNA names mentioned in the original articles. We compared the coordinates of lncRNAs against Ensembl annotations and considered identity if the two lncRNAs highly overlap (i.e. >90%). To facilitate linking to other databases, we provided the Ensembl identifiers (18), Entrez ID and alias names for the lncRNAs based on lncRNA names and coordinates. Finally, we used a standardized classification scheme, the Disease Ontology (19), to annotate human diseases. We also characterized the expression dynamics of functional lncRNA–target regulations using the RNA-seq data of ∼11 000 patients in pan-cancers from The Cancer Genome Atlas (TCGA) (20). Using fastq files as input, kallisto (version 0.43.0) (21) was used to align the reads to the GENCODE reference transcriptome (version 24) (22) and quantify transcript-expression levels in transcripts per million (TPM). We downloaded these data from the open-access repository (https://osf.io/gqrz9) (23). Differential analysis of the RNA-seq data was performed using the DESeq2 package. Gene expression changes were considered statistically significant when their fold change with a cutoff of 2 and FDR <0.05. Pearson correlation coefficients between lncRNAs and key targets in TCGA cancer samples were calculated to reveal the dynamic expression correlation. A *t*-test of the Pearson correlation coefficient was used to estimate the correlation significance. Finally, the disease-associated lncRNA–target regulations and all the related information were loaded into the MySQL database management system. Figure 1 schematically illustrates the general workflow and features of the database.

## DATABASE STATISTICS

As of December 2018, the current version of LncTarD consists of 2822 key lncRNA–target regulations in 177 human diseases, including 475 lncRNAs, 391 miRNAs, 774 protein-coding genes and 140 biological functions. In addition, LncTarD records 1613 experimentally-supported functional regulations including ceRNA or sponge (840 entries), transcriptional regulation (169 entries), epigenetic regulation (255 entries), interaction with protein (326 entries), interaction with mRNA (30 entries), and chromatin looping (3 entries) and 1209 expression associations in human diseases. For example, there are 128 lncRNA-PRC2 interactions involving 63 lncRNAs (such as HOTAIR, MALAT1, TUG1, PVT1, HOXA11-AS, CHKN2B-AS1, UCA1, H19, XIST and SNHG6) in 39 human diseases, including gastric cancer, breast cancer, colorectal cancer, hepatocellular carcinoma and non-small cell lung cancer. Recent studies have shown that the interactions between lncRNAs and PRC2 has potential therapeutic implications (14). The metastatic potential of breast cancer can be limited by preventing the interaction of HOTAIR with PRC2 or LSD1 complexes using small molecule inhibitors (24,25). The precise molecular mechanisms of lncRNA actions in human diseases are necessary for the exploration of these potential new avenues in cancer therapy.

**Figure 2.**
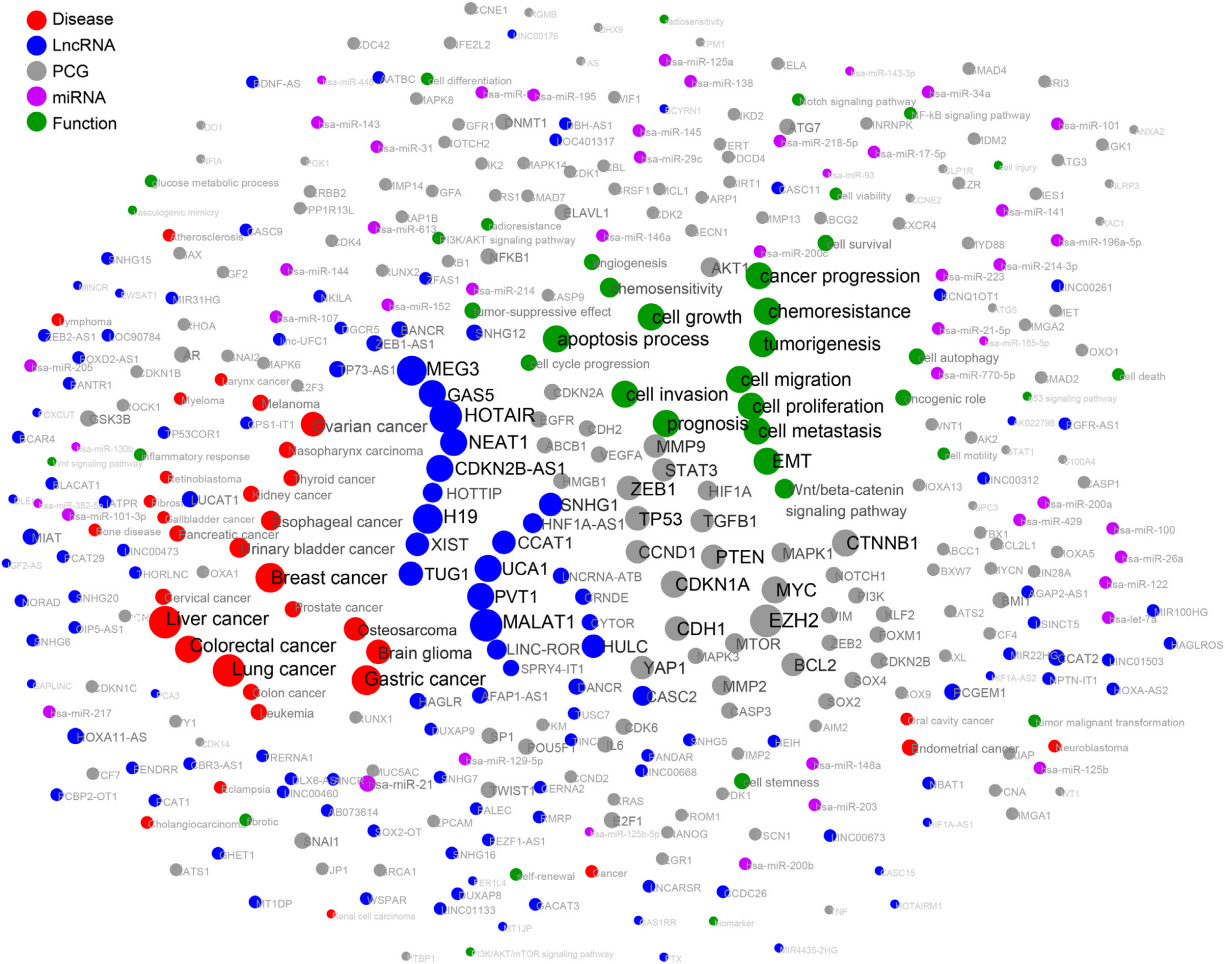
Network of lncRNA–target regulations, functions, and diseases. Only nodes with degrees >5 are shown. Red circle represents disease, blue circle represents disease-associated lncRNA, gray circle represents key target gene, green circle represents influenced biological function, and purple circle represents miRNA. The lines correspond to experimentally-supported associations. The size of the nodes and labels corresponds to their degree.

Utilizing the data in LncTarD, a network of diseases, lncRNA–target regulations and biological functions were constructed (Figure 2). The top 10 key lncRNAs ranked by the number of associated lncRNA–target regulations include MALAT1 (257 entries), HOTAIR (248 entries), H19 (131 entries), MEG3 (102 entries), UCA1 (83 entries), PVT1 (75 entries), CDKN2B-AS1 (74 entries), GAS5 (61 entries), NEAT1 (60 entries), and TUG1 (58 entries). The top 10 diseases ranked by the number of associated lncRNA–target regulations include lung cancer (320 entries), liver cancer (300 entries), gastric cancer (233 entries), breast cancer (191 entries), colorectal cancer (159 entries), brain glioma (148 entries), ovarian cancer (138 entries), osteosarcoma (122 entries), esophageal cancer (114 entries), and urinary bladder cancer (99 entries), which are represented by 64.6% of the curated disease-associated regulations. We observed that 31.4% (188/598) of the regulators and 32.1% (333/1039) of the targets frequently occur in multiple types of disease. For example, expression dysregulation of HOTAIR has been observed in 42 human diseases, such as breast cancer, ovarian cancer, hepatocellular carcinoma, lung cancer, and atherosclerosis. Analogously, expression dysregulation of TP53 has been observed in 22 human diseases, such as breast cancer, non-small cell lung cancer, malignant glioma, cardiac fibrosis and pulmonary hypertension. However, 11.9% (282/2372) of the lncRNA–target regulations were found to be present in diverse types of disease. Most of the disease-associated lncRNA–target regulations (88.1%, 2090/2372) were restricted to one disease type, suggesting the high specificity of lncRNA-mediated gene regulation.

**Figure 3.**
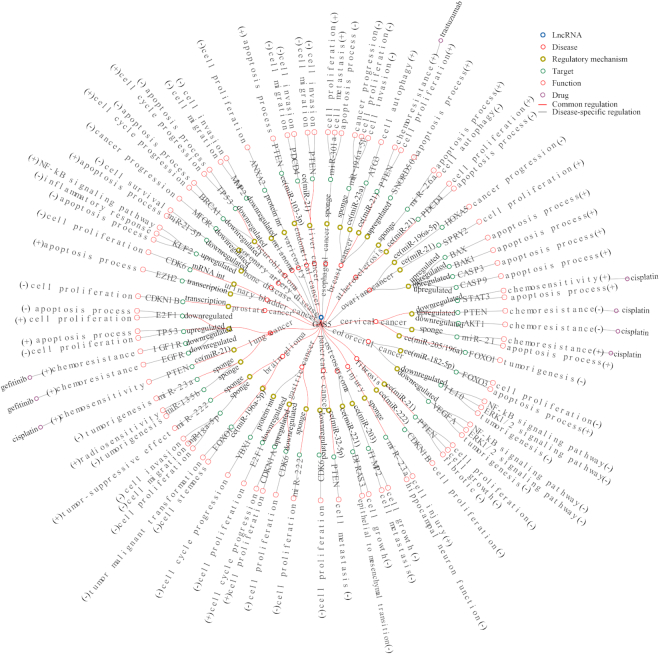
Network of GAS5-mediated regulations, regulatory mechanisms, functions, drugs and diseases. Blue circles represents GAS5, red circles represent disease, yellow circles represent GAS5-mediated regulatory mechanisms, green circles represent key target genes, light red circles represent influenced biological functions, and purple circles represent drugs. Red lines correspond to common regulations in at least two diseases. Gray lines correspond to disease-specific regulations. GAS5-mediated regulatory mechanisms in several diseases have not been discovered in the current literatures. We recorded their expression associations (upregulated or downregulated). Abbreviations: ce (ceRNA), transcription (transcriptional regulation), protein int (interacting with proteins), and mRNA int (interact with mRNA).

This network reveals that some key lncRNAs, including MALAT1, HOTAIR, H19, MEG3, UCA1, PVT1 and GAS5, affect certain important biological functions (such as cell proliferation, cell invasion, cell migration, cell apoptosis, and cell metastasis) by regulating downstream key mediators (such as well-known cancer genes EZH2, CDKN1A, CDH1, MYC, ZEB1, TP53, CTNNB1, CCND1, PTEN and BCL2). For example, GAS5 is differentially expressed in 22 human diseases and regulates the downstream key mediator PTEN to affect cell proliferation, cell invasion, cell migration, and chemoresistance (cisplatin and trastuzumab) in seven human diseases. Among 85.7% (6/7) of these diseases, GAS5 acts as a molecular sponge for miRNAs, such as miR-21 in cardiac fibrosis (26), non-small cell lung cancer (27), her2-receptor positive breast cancer (28), and hepatocellular carcinoma (29); miR-32-5p in pancreatic cancer (30), and miR-103-3p in endometrial cancer (31), to reduce their inhibitory effects on PTEN (Figure 3). However, 57.1% (32/56) of GAS5-mediated regulations are disease-specific, as illustrated by the finding that GAS5 regulates cell proliferation, cell migration, and cell invasion by sponging miR-18a-5p in glioma (32) but sponging miR-196a-5p in triple-receptor negative breast cancer (33). We found that GAS5-mediated explicit regulatory mechanisms in several diseases, including bone disease, melanoma and coronary artery disease, had not been discovered in the current literature. We annotated both positive and negative associations between the expression levels of disease-associated lncRNA and associated target genes. These common lncRNA-mediated regulations in diverse diseases may represent a similar underlying mechanism in the pathogenesis of disease. Thus, these disease-specific lncRNA-mediated gene regulations may play an important role in understanding the regulatory mechanisms of disease heterogeneity.

## WEB INTERFACE

**Figure 4.**
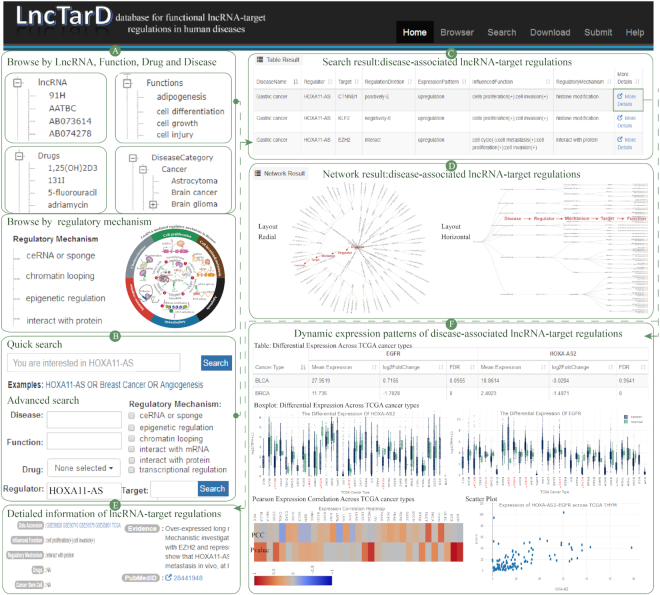
Schematic workflow of LncTarD. (**A**) Interface of the browse module; (**B**) interface of the quick search and advanced search modules, with HOXA11-AS1 as an example; (**C**) search result page for HOXA11-AS1; (**D**) network result of lncRNA–target regulations in human diseases; (**E**) search result page with detailed information; (**F**) interface of dynamic expression patterns of disease-associated lncRNA–target regulations across 33 TCGA cancer types.

LncTarD is publicly available at http://biocc.hrbmu.edu.cn/LncTarD/ and http://bio-bigdata.hrbmu.edu.cn/LncTarD. LncTarD provides a user-friendly Web interface that allows intuitive browsing, searching, visualization, and retrieval of all disease-associated lncRNA–target regulations in the database (Figure 4). From the ‘Browse’ page, users can browse all experimentally-supported disease-associated lncRNA–target regulations by selecting the lncRNA symbol, lncRNA-mediated regulatory mechanisms, influenced biological functions, and disease names in a pull-down menu (Figure 4A). Following their selection, a list of matched entries is returned. The Browse page provides the statistics of disease-associated lncRNA–target regulations. On the ‘Search’ page, ‘Advanced Search’ provides two search panels for searching experimentally-supported functional regulation and expression association, respectively. Search panels provide multiple options for users to create customized searches of descriptions containing the disease, lncRNA-mediated regulatory mechanism, function, drug, lncRNA, and target gene of interest. The home page of the website provides a ‘Quick search’ utility which can be used to query the database for the lncRNA and function of interest, as well as the disease name (Figure 4B). For instance, searching for lncRNA ‘HOXA11-AS1’ will return related entries. The query result is presented as a responsive table, which contains the basic information of disease-associated lncRNA–target regulations, including the lncRNA and target gene, associated disease, regulatory direction, dysregulated expression pattern of lncRNAs, and associated biological functions, as well as lncRNA-mediated regulatory mechanisms (Figure 4C). A network (three types of layout) is provided to intuitively display the ways in which these disease-related lncRNAs contribute to pathogenesis, including the downstream key targets, affected functions, regulatory mechanisms of lncRNAs, and related drugs in human diseases (Figure 4D). By clicking the ‘More details’ link for a specific disease-associated lncRNA–target, LncTarD lists more detailed annotation information, including download of lncRNA microarray or lncRNA sequence data, associated drug, cancer hallmarks, evidence, and links to the reference (Figure 4E). Differentially-expressed patterns and dynamic expression correlations of the functional lncRNA–target regulations using lncRNA microarray, lncRNA sequence data and 33 TCGA cancer types are provided (Figure 4F). In addition, on the ‘Help’ page, LncTarD provides a detailed tutorial for the usage of the database. The full dataset is available as a tab-delimited file on the ‘Download’ page of the website. Submission of new or updated information based on peer-reviewed publications can be made on the ‘Submit’ page.

## CONCLUSIONS AND FUTURE EXTENSIONS

In this study, we developed a database that attempts to collect and illustrate the experimentally-verified lncRNA–target regulations, their influenced functions, and lncRNA-mediated regulatory mechanisms in human diseases. LncTarD provides: (i) key downstream targets and important biological functions (such as cell proliferation, EMT, and angiogenesis) driven by disease-related lncRNAs in human diseases; (ii) lncRNA-mediated regulatory mechanisms in human diseases, including transcriptional regulation, epigenetic regulation, chromatin looping, ceRNA or sponge, interaction with mRNA, and interaction with proteins; (iii) the association between the expression levels of disease-associated lncRNA and associated target genes, which would be useful in further-investigation; (iv) lncRNA–target regulations responsible for drug resistance or sensitivity involving 52 drugs in 41 human diseases, with the goal of providing potential lncRNA–targeted strategies for overcoming drug resistance in cancer therapy and (v) all lncRNA microarray, lncRNA sequence data and TCGA pan-cancer transcriptome data (33 cancer types), which were also integrated into LncTarD to help characterize the dynamics of functional lncRNA–target regulations. LncTarD provides a user-friendly interface to search, browse, and visualize information concerning these disease-associated lncRNA–target regulations.

In the future, with the increasing amount of new high-throughput multi-omics data derived from patients with different diseases, we will characterize the effects of lncRNA–target regulations on human diseases in terms of the following aspects: (i) lncRNAs can regulate gene expression through histone modification, DNA methylation, and chromatin structure in human diseases. Histone modification datasets (ChIP-seq) and DNA methylation datasets (such as WGBS, RRBS-seq, MeDIP-Seq, and methylation array) as well as long-range promoter-enhancer interactions with high-resolution capture experiments (such as Hi-C, ChIA-PET and 5C) in human diseases will be integrated into LncTarD. (ii) lncRNAs can interact with specific proteins to participate in disease progression by regulating protein activity and function. RNA-RNA and protein-RNA interactions with RIP-seq and CLIP-Seq in human diseases will be integrated into LncTarD. We hope that the integration of multi-omic data for diseases will help expand coverage of the genome, which would enable the discovery of disease-related lncRNAs in disease progression. Furthermore, we will regularly update and improve the database and add more annotation information and practical analysis tools to keep pace with ongoing research. We believe that LncTarD can serve as a timely and valuable resource for the further understanding of the functions and molecular mechanisms of lncRNA deregulation in disease pathogenesis, which will help to identify novel and sensitive biomarkers and therapeutic targets in human diseases.

## Supplementary Material

gkz985_Supplemental_File
